# Loss-of-function mutations in *MYO15A* and *OTOF* cause non-syndromic hearing loss in two Yemeni families

**DOI:** 10.1186/s40246-023-00489-1

**Published:** 2023-05-15

**Authors:** Maria Asaad, Mona Mahfood, Abdullah Al Mutery, Abdelaziz Tlili

**Affiliations:** 1 Department of Applied Biology, College of Sciences, University of Sharjah, Building W8 - Room 107, P.O. Box 27272, Sharjah, UAE; 2 Human Genetics and Stem Cells Research Group, Research Institute of Sciences and Engineering, University of Sharjah, Sharjah, UAE

**Keywords:** Non-syndromic hearing loss, Frameshift mutation, Splice site mutation, *MYO15A* gene, *OTOF* gene, Clinical exome sequencing, Target enrichment panel

## Abstract

**Background:**

Hearing loss is a rare hereditary deficit that is rather common among consanguineous populations. Autosomal recessive non-syndromic hearing loss is the predominant form of hearing loss worldwide. Although prevalent, hearing loss is extremely heterogeneous and poses a pitfall in terms of diagnosis and screening. Using next-generation sequencing has enabled a rapid increase in the identification rate of genes and variants in heterogeneous conditions, including hearing loss. We aimed to identify the causative variants in two consanguineous Yemeni families affected with hearing loss using targeted next-generation sequencing (clinical exome sequencing). The proband of each family was presented with sensorineural hearing loss as indicated by pure-tone audiometry results.

**Results:**

We explored variants obtained from both families, and our analyses collectively revealed the presence and segregation of two novel loss-of-function variants: a frameshift variant, c.6347delA in *MYO15A* in Family I, and a splice site variant, c.5292-2A > C, in *OTOF* in Family II. Sanger sequencing and PCR–RFLP of DNA samples from 130 deaf and 50 control individuals confirmed that neither variant was present in our in-house database. In silico analyses predicted that each variant has a pathogenic effect on the corresponding protein.

**Conclusions:**

We describe two novel loss-of-function variants in *MYO15A* and *OTOF* that cause autosomal recessive non-syndromic hearing loss in Yemeni families. Our findings are consistent with previously reported pathogenic variants in the *MYO15A* and *OTOF* genes in Middle Eastern individuals and suggest their implication in hearing loss.

## Background

Hearing loss (HL) is a common sensory deficit with diverse clinical symptoms and forms. In most cases, hearing loss is caused by genetic factors. Non-syndromic hearing loss is the predominant form of hereditary hearing loss worldwide, accounting for up to 70% of all hearing loss cases. In this form, hearing impairment is the sole clinical manifestation. Approximately 75–80% of mutant alleles responsible for non-syndromic hearing loss have an autosomal recessive inheritance pattern, resulting in autosomal recessive non-syndromic hearing loss (ARNSHL) [1]. Although ARNSHL is mostly monogenic in nature and follows Mendelian inheritance patterns, it remains a challenging condition to diagnose and screen [2]. With 78 genes and over 90 loci identified, ARNSHL exhibits clinical and genetic heterogeneity (https://hereditaryhearingloss.org/).

The genetic heterogeneity of ARNSHL, or hearing loss in general, is attributed to the diversity of specialized cell types found in the complex auditory system and their expression of a considerable number of distinct proteins, which in turn associate with one another, to orchestrate the physiological process of hearing [3, 4]. Among these crucial proteins are the myosin proteins, which are involved in cellular organization [5]. Myosins are generally known as actin-based motor proteins that utilize ATP hydrolysis to facilitate intracellular trafficking of a large variety of cargo, maintain mitotic spindle formation during cellular division, and generate force in cells [6]. The *MYO15A* gene encodes for unconventional myosin XVa (3230 amino acids) (UniProt ID: Q9UKN7) and is comprised of 66 exons that span 71 Kb on chromosome 17p11.2. Its association with non-syndromic hearing loss was first reported following positional and functional cloning strategies that were undertaken to identify the causative gene at the autosomal recessive non-syndromic deafness (DFNB3) locus (OMIM 600,316) mapped in affected individuals from an isolated village in Bali, and two unrelated families from India, in addition to the *shaker-2* (*sh2*) locus implicated in deafness and vestibular dysfunction in the *sh2* mouse [7]. Due to conserved synteny and the presence of a murine orthologue for the causative gene, the critical interval was further narrowed down from 3.0 to 0.2 cM [8]. Subsequently, Probst FJ et al. reported the phenotypic rescue of hearing in a *sh2* mouse after being injected with constructed artificial bacterial chromosomes (BACs) comprising the refined critical interval and ultimately identified the *MYO15A* gene from cDNA samples [9].

Another subcategory of proteins is involved in neural transmission, including otoferlin and pejvakin [5]. The *OTOF* gene encodes for the otoferlin protein (1997 amino acids) (UniProt ID: Q9HC10) and comprises of 46 exons that span 90 Kb on chromosome 2p23.3. The implication of the *OTOF* gene in non-syndromic recessive deafness DFNB9 (OMIM 601,071) was first reported by Yasunaga et al. in four unrelated consanguineous Lebanese families by combining the candidate gene approach with positional cloning [10]. One year later, Yasunaga et al. reported a splice site mutation in the *OTOF* gene in a consanguineous Indian family with DFNB9 [11].

*MYO15A* and *OTOF* were among the first genes identified to be associated with deafness using conventional approaches [12]. Currently, they are among the top contributors to non-syndromic recessive deafness with varying frequencies among different populations, including those in the Middle East [13–15]. The exploitation of next-generation sequencing (NGS) technologies has led to an exponential increase in the identification rate of genes and variants of genetically heterogeneous conditions, including hereditary hearing loss; greatly contributing to a large portion of our current knowledge of the genetics of non-syndromic hearing loss [2]. In accordance with this statement, the genetic burdens of the *MYO15A* and *OTOF* genes have been made more comprehensive with the utilization of NGS. Presently, 220 and 375 pathogenic/likely pathogenic variants have been reported in the *OTOF* and *MYO15A* genes, respectively (http://deafnessvariationdatabase.org/).

In the present study, two unrelated consanguineous Yemeni families segregating ARNSHL were screened using clinical exome sequencing (CES) to identify the causative variants. Our findings identified a novel frameshift variant and a splice site variant in *MYO15A* and *OTOF* genes, respectively. The association of the identified pathogenic variants with ARNSHL highlights the essential role of these two protein-coding genes in the complex auditory system.

## Methods

### Subjects and clinical assessment

Two unrelated Yemeni families, each with two affected individuals, were recruited from the Al-Amal Association of Deaf and Mutism (Mukallah, Yemen) to conduct this study. Genomic DNA was extracted from the collected saliva samples using the Oragene-DNA (OG-500) Kit (DNA Genotek, Canada), and labelled with codes to ensure subjects’ confidentiality. In addition, DNA samples of 130 unrelated affected individuals and 50 hearing controls from the same demographic region were collected and included in this study. Family history was recorded for both families, and affected individuals underwent physical and clinical assessments, including pure-tone audiometric tests.

### *GJB2* screening

**Fig. 1:**
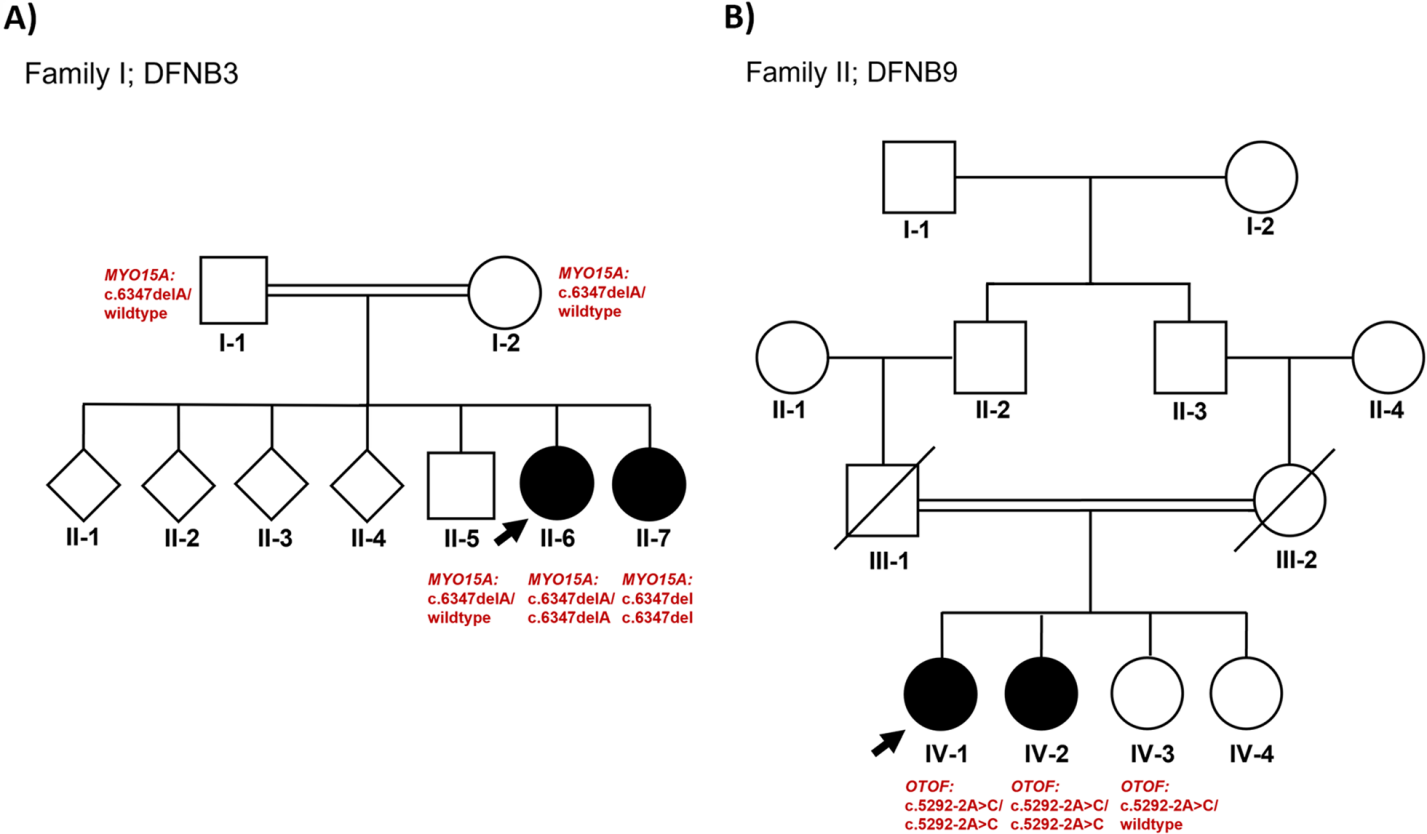
Pedigrees of families I and II. **A** Pedigrees of Family I segregating DFNB3, and **B** Family II segregating DFNB9. Genotypes are indicated in red for each individual. Arrows denote the probands

Affected individuals from families I (II-6 and II-7) and II (IV-1, IV-2) (Fig. 1) underwent an initial screening to identify any variants in the *GJB2* gene, as mutations in this gene constitute up to half of all hereditary hearing loss cases in many populations including those in the Middle East [16]. In brief, PCR products corresponding to the coding region of the *GJB2* gene (exon 2) were amplified from the genomic DNAs of the affected family members using Cx26-2F and Cx26-2R primers (Table 1). PCR amplicons were then purified using ExoSAP-IT™ PCR Product Cleanup Reagent (Affymetrix, Fisher Scientific, Göteborg—Sweden) and cycle sequenced using the Big Dye Terminator V3.1 Cycle Sequencing Kit (Applied Biosystems, USA). Cycle sequencing products were then purified by EDTA/sodium acetate/ethanol precipitation and injected into the 3500 Genetic Analyzer (Applied Biosystems, Thermo Fisher Scientific, USA). Finally, the generated sequences were aligned with the publicly available wild-type sequence of the *GJB2* gene (NM_004004.6) using the Basic Local Alignment Search Tool (BLAST) (https://blast.ncbi.nlm.nih.gov/Blast.cgi).

**Table 1:** Primers used in this study

Primer name	Sequence (5′-3’)
*Cx26_2F* *Cx26_2R* *MYO15A_Ex30_F*	ACACGTTCAAGAGGGTTTGG GGGAAATGCTAGCGACTGAG CATGAGAGGGATGCATGTTGTG
*MYO15A_Ex30_R*	CACTCTGAGTTCTGGAAGTCCC
*OTOF_Ex43_F*	CTTCTTCACAGGGGAGAAGTC
*OTOF_Ex43_R*	TCTGATGGACTGGAAGCAATA

### Clinical exome sequencing and bioinformatic analysis

Genomic DNA of the proband in each family was subjected to CES using a NGS screening panel that targets 8,000 clinically relevant genes. In brief, genomic DNA was sheared and end-repaired DNA fragments were ligated to adapters for library preparation. Exome capture and target enrichment were then performed using the SureSelect Clinical Research Exome V2 kit (Agilent Technologies, Santa Clara, CA), and pair-end sequencing was performed using the Illumina HiSeq 2500 system (Illumina, San Diego, CA, USA). Raw reads were then sorted into high-quality reads and mapped to the human reference genome (GRCh37/hg19) using the Burrows–Wheeler aligner (BWA). Duplicate reads were marked and sorted using Picard tools, and variants were called using Genomic Analysis Toolkit (GATK). Called variants were then annotated using the in-house Variation and Mutation Annotation Toolkit (VariMAT v2.4.1) to aid the process of variant interpretation and subsequent prioritization. The Ensembl and RefSeq transcript sets were used to annotate genome information and perform the functional assessment of all on-target variants by integrating clinical grade databases (GWAS, ClinVar, etc.), variant class prediction and variant pathogenicity databases and algorithms. Annotations that rely on the Ensembl release 87 VEP program included population frequencies (ExAC, dbSNP, 1000Genome databases, etc.) and computational pathogenicity predictions (SIFT, PolyPhen-2, Condel, etc.) [17]. Transcript-specific predicted outcomes of non-synonymous SNVs were also integrated from the dbNSFP v4 database and included pathogenicity predictions and conservation scores [18, 19]. Variant annotation was then followed by filtration according to the following criteria: (A) variants with allelic frequencies < 0.5% or absent in the ExAC Browser, gnomAD, 1000Genomes, and dbSNP databases, (B) frameshift, splice site, stop-gained, and missense variants, (C) homozygous variants (D) variants in ARNSHL-associated genes (https://hereditaryhearingloss.org/).

Ultimately, the following in silico tools: Mutation Taster, (https://www.mutationtaster.org/), SIFT (https://sift.bii.a-star.edu.sg/), VEP (https://asia.ensembl.org/Tools/VEP), PolyPhen-2 (http://genetics.bwh.harvard.edu/pph2/), Human Splicing Finder (http://www.umd.be/HSF3/), and SpliceAI (https://spliceailookup.broadinstitute.org/), were used to predict the impact of the candidate variants obtained by CES data analysis.

### Sanger sequencing

To verify the candidate variants obtained from CES data analysis and confirm their co-segregation with the hearing loss phenotype in each respective family, Sanger sequencing of the regions where the variants are located was performed for the recruited members of both families. In brief, PCR primers corresponding to exon 30 and exon 43 of the *MYO15A* and the *OTOF* genes were designed using Primer 3, respectively (http://www.bioinformatics.nl/cgi-bin/primer3plus/primer3plus.cgi). The primers used are listed in Table 1. PCR products were amplified from genomic DNAs using the above-mentioned primers, purified using ExoSAP-IT™ PCR Product Cleanup Reagent (Affymetrix, Fisher Scientific, Göteborg—Sweden) and cycle sequenced using the Big Dye Terminator V3.1 Cycle Sequencing Kit (Applied Biosystems, USA). Cycle sequencing products were subsequently purified by EDTA/sodium acetate/ethanol precipitation and injected into the 3500 Genetic Analyzer (Applied Biosystems, Thermo Fisher Scientific, USA). DNA samples of 130 unrelated affected individuals and 50 hearing controls were also sequenced for the *MYO15A* gene, as mentioned above, to screen for the presence of the *MYO15A* variant. All sequences generated were viewed on the Sequencing Analysis Software v6.0 and aligned with their respective reference sequences of the *MYO15A* (NM_016239.4) or the *OTOF* (NM_001287489.2) genes using BLAST.

### PCR–RFLP

The candidate *OTOF* variant was screened in 130 unrelated affected individuals and 50 hearing controls using the alternative approach of PCR–RFLP, as the variant introduces an AgeI restriction site in the corresponding PCR product. In brief, PCR products were generated from the genomic DNAs of individuals using the *OTOF* primers (Table 1) and then enzymatically digested with AgeI according to the manufacturer’s protocol (Thermo Fisher Scientific, Waltham, USA). The digested products were then separated by 2% agarose gel electrophoresis.

## Results

### Phenotype characterization

Two unrelated Yemeni families, each with two affected deaf individuals, were recruited for this study (Fig. 1). Family history confirmed consanguinity in each family and suggested an autosomal recessive mode of inheritance. In addition, physical and pure-tone audiometric tests ruled out any other clinical abnormalities and confirmed the phenotype of sensorineural hearing loss in the affected individuals in both families.

### Clinical exome sequencing data analysis and in silico prediction analysis

**Fig. 2:**
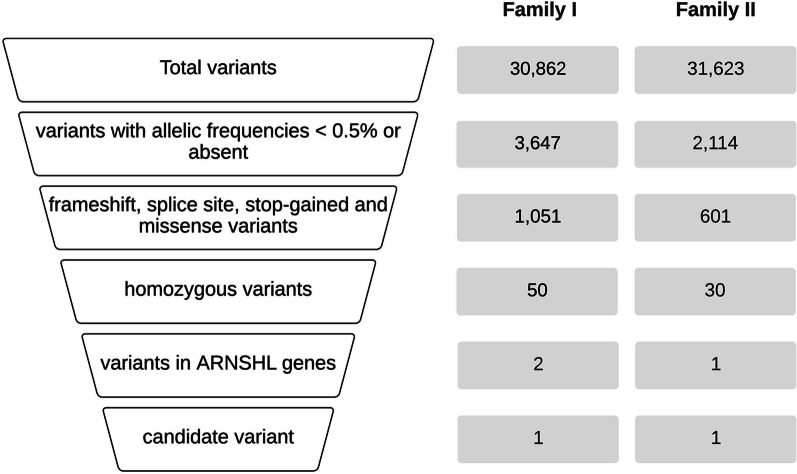
Variant prioritization steps followed in this study

Following preliminary *GJB2* screening via Sanger sequencing, the affected individuals were negative for *GJB2* mutations. Thus, CES was performed to investigate the ARNSHL causative variants segregating in each family. The variant prioritization steps and the corresponding number of variants at each filtration step are shown in Fig. 2.

**Fig. 3:**
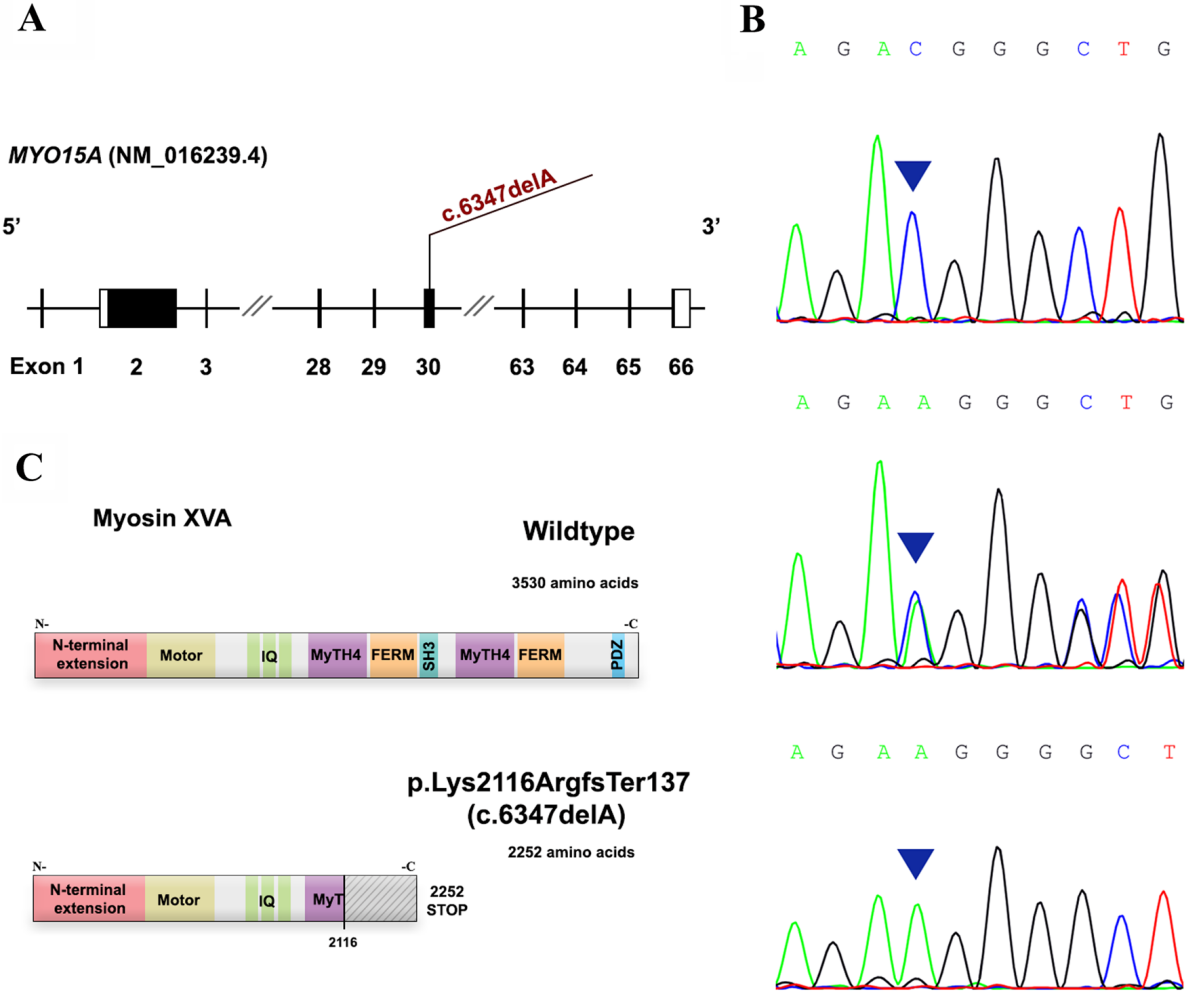
*MYO15A* gene structure, electropherograms, and in silico analysis. **A** The location of the identified variant with respect to the *MYO15A* gene. Solid rectangles represent coding regions. **B** Electropherograms of a homozygous mutant profile (top panel), a heterozygous profile (middle panel), and a homozygous wild-type profile (bottom panel) for the c.6347delA variant in the *MYO15A* gene. **C** pathogenicity predicted impact of the c.6347delA variant at protein level. IQ: Calmodulin-binding motif. MyTH4: Myosin Tail Homology 4. FERM: (4.1, ezrin, radixin, and meosin). *SH3* SRC Homology 3. *PDZ* Post-synaptic density protein

In Family I, our analysis revealed the presence of two variants in the *MYO15A* (NM_016239.4) gene: the first variant c.6347delA, p.Lys2116ArgfsTer137, a homozygous deletion of adenine (Fig. 3A), and the second variant c.6348G > C, a homozygous G to C transversion. We determined the impact of both variants and found c.6347delA to be “disease-causing” and “IMPACT:HIGH” using Mutation Taster and VEP, respectively. However, the impact of c.6348G > C was predicted to be “tolerated” by SIFT and “probably damaging” by PolyPhen-2.

**Fig. 4:**
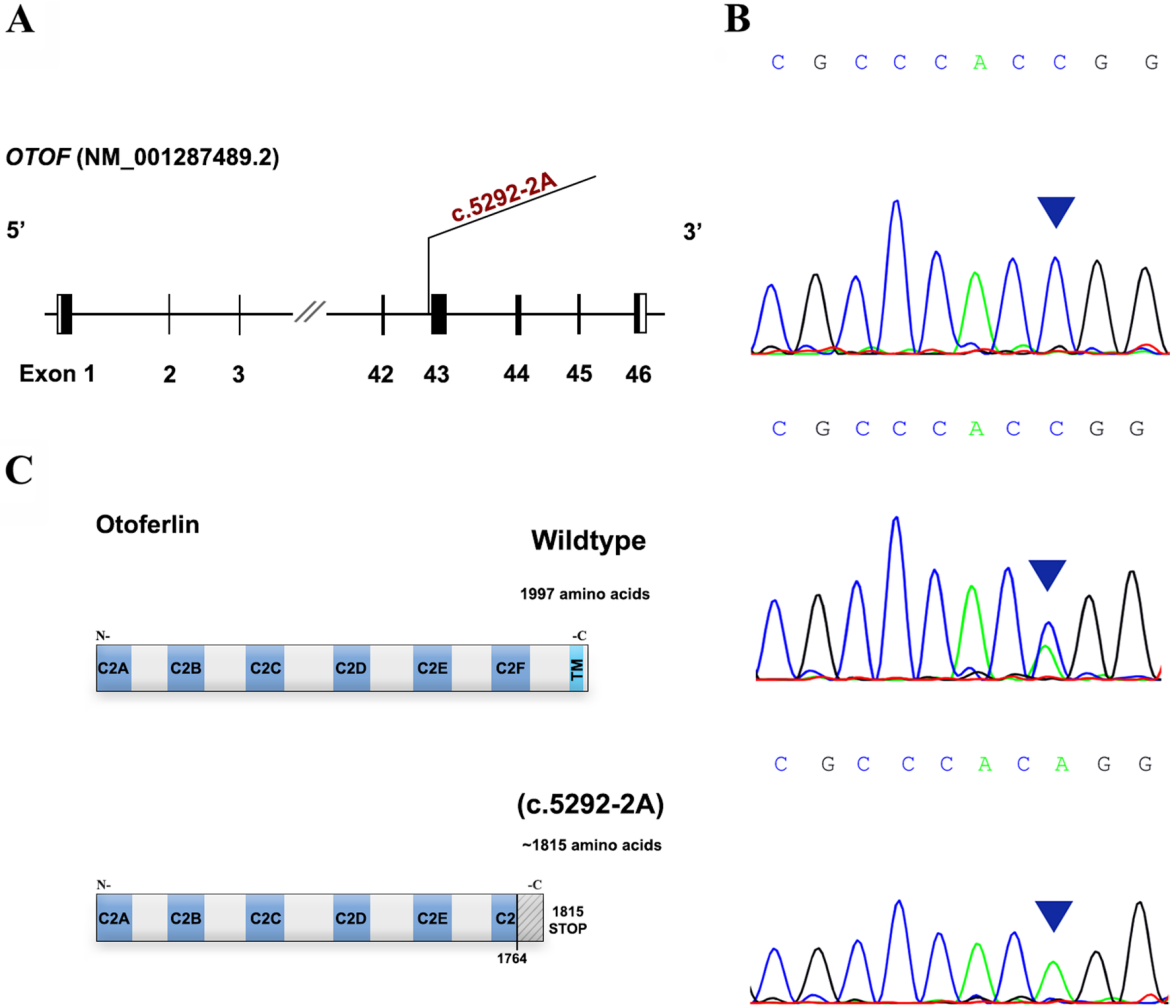
*OTOF* gene structure, electropherograms, and in silico analysis. **A** The location of the identified variant with respect to the *OTOF* gene. Solid rectangles represent coding regions. **B** Electropherograms of a homozygous mutant profile (top panel), a heterozygous profile (middle panel), and a homozygous wild-type profile (bottom panel) for the c.5292-2A > C variant in the *OTOF* gene. **C** Pathogenicity predicted impact of the c.5292-2A > C variant at protein level. C2: Ca2 + -binding domain

In Family II, using the same filtration strategy, we identified the presence of a homozygous splice site variant c.5292-2A > C in the *OTOF* (NM_001287489) gene (Fig. 4A). The A > C transversion was predicted to disrupt the invariant AG sequence of the acceptor site preceding exon 43 in the *OTOF* gene, using the Human Splicing Finder and SpliceAI tools.

### Sanger sequencing and PCR–RFLP

The candidate *MYO15A* variant c.6347delA was validated from the CES data using Sanger sequencing. We found this variant in segregation with the hearing loss phenotype in Family I (Fig. 3B). Both the affected sisters (II-6 and II-7) shared homozygosity for the variant. However, their clinically unaffected brother (II-5) and parents were heterozygous for this variant. Moreover, Sanger sequencing analysis revealed that this *MYO15A* variant was absent in 130 unrelated affected individuals and 50 hearing controls. However, the second *MYO15A* variation, c.6348G > C, was found in the homozygous state in all the screened family members.

The presence of the candidate *OTOF* variant c.5292-2A > C was confirmed by Sanger sequencing. In addition, our segregation analysis showed that it was indeed in segregation with the hearing loss phenotype in Family II (Fig. 4B). Both the affected sisters (IV-1 and IV-2) were homozygous for the variant, whereas their clinically unaffected sister (IV-3) was heterozygous. In addition, this splice site variant was not found in 130 unrelated affected individuals and 50 hearing controls following our PCR–RFLP analysis.

## Discussion

In this study, we recruited two consanguineous Yemeni families with hereditary hearing loss. Families I and II were presented with severe-to-profound sensorineural hearing loss. All affected individuals were found to be *GJB2*-negative, allowing us to exclude the predominantly implicated gene in deafness in many populations [20, 21] The CES approach we have then utilized along with the appropriate variant prioritization strategy revealed two candidate variants in the *MYO15A* and the *OTOF* genes.

The myosin superfamily is divided into conventional myosins (class-II), primarily involved in muscle contraction, and unconventional myosins (at least 14 classes). Unconventional myosins share the head-motor domain and the neck region as their “conventional” counterpart. However, they are characterized by the presence of divergent tails that provide structural and functional features that render them intracellularly multifunctional. Dysfunction of different myosin proteins has been linked to various clinical conditions, including blindness, neurological pathologies and kidney disease [6]. Therefore, despite the abundance of myosins, the reported organ-specific pathologies suggest that some, if not all, classes of myosins are crucial and non-redundant in certain organs [22]. Likewise, in the human auditory system, unconventional myosins play an indispensable role in the physiological process of hearing. *MYO15A* is among three other unconventional myosins (*MYO3A*, *MYO6*, and *MYO7A)*, which have been previously linked to different forms of DFNB [23]. Previous studies have shown that myosin XVa colocalizes at the stereocilia tips of sensory hair cells [24] and is required to form a molecular complex by trafficking Whirlin (*WHRN*) and Eps8 (*EPS8;* epidermal growth factor receptor pathway substrate 8) to stereocilia tips [22, 25]. Using *MYO15A* isoform-specific knockout mice, it was also shown that the two distinct functions of myosin XVa are isoform-specific: Isoform 1 (396 kDa) is involved in the assembly of stereocilia, and isoform 2 (262 kDa) is involved in stereocilia maintenance [26]. Therefore, the absence of *MYO15A* not only diminishes the characteristic staircase architecture of stereocilia in sensory hair cells but also prevents their long-term maintenance, both of which are essential for the functionality of sensory epithelial cells.

Remarkably, Zhang et al. reported that the number of homozygous *MYO15A* variants was the highest in deaf individuals from the Middle East, a trend that is attributed to the deeply rooted practice of consanguineous marriages within the region [13, 27]. In Yemen in particular, the consanguinity rates have been estimated to be between 39.9 and 44%. [28, 29]. In addition to the relatively high diagnostic rate of *MYO15A* variants in Middle Eastern populations, recurrent mutations with a founder effect have also been reported, such as p.Y393Cfs*41 in 30% (8/26) of Omani patients [30].

The frameshift variant c.6347delA, p.Lys2116ArgfsTer137, would lead to the substitution of the amino acid lysine encoded by codon 2116, ultimately shifting the open reading frame of the *MYO15A* gene and introducing a premature termination codon (PTC). As a result, a truncated protein (2252 amino acids), which predictably triggers nonsense-mediated decay (NMD) or RNA degradation, would be translated (Fig. 3C). In addition, this mutation resides in the tail region of the protein, which is critical for many protein–protein interactions [23]. The N-terminal region consists of two MyTH4-FERM domains (myosin tail homology 4-band 4.1, ezrin, radixin, and meosin) repeats, separated by a SH3 (SRC homology 3) domain, all of which would no longer be part of the synthesized truncated protein. Although pathogenic variants in almost all coding exons of the *MYO15A* gene have been reported, most are found in the motor domain or the MyTH4/FERM domains [13]. Finally, mutations affecting the tail region of myosin VII (UniProt ID: Q13402), which shares structural similarity with myosin XVa, have also been linked to DFNB2, DFNA11 and USH1B [31].

The frameshift mutation NM_016239.4 (*MYO15A*): c.6347delA also met the criteria of PVS1_Strong, PM2_Strong and PP1_supporting for an overall classification as “pathogenic” based on the American College of Medical Genetics and Genomics/Association for Molecular Pathology (ACMG/AMP) guidelines. Collectively, segregation of the c.6347delA variant with the disease phenotype and our in silico predictions confirm the pathogenicity of this variant in Family I.

As for the second variant c.6348G > C found in Family I, segregation of the variant in both affected and unaffected members of the family led us to rule out its pathogenicity and classify it as a polymorphic variant. The missense variation showed conflicting predictions using bioinformatics tools. However, SIFT predicted the substitution of the lysine residue to asparagine at position 2116 to be “tolerated” which was in line with our findings. Given that this variant has not been previously reported in online databases, its allelic frequency remains unknown and may be determined by screening controls from the same region.

Otoferlin is a type II transmembrane protein comprised of six (or seven) C2 domains that mediate calcium and phospholipid binding events that are essential for membrane fusion and trafficking. In the auditory system, this protein is expressed in sensory hair cells and is crucial for Ca^2+^-mediated synaptic vesicle exocytosis. The absence of this protein prevents the release of neurotransmitters to type I spiral ganglion neurons (SGNs) and thus withholds sound perception. This is supported by observations made by Roux et al., as *Otof* -/- mice are deaf and deficient in synaptic exocytosis [32]. Restoration of the otoferlin protein in *Otof* -/- mouse models has also proven its crucial role, particularly in early inner hair cell (IHCs) synapse maturation [33]. Although mutations in the *OTOF* gene are not major contributors to hearing loss in Iran—the second largest country in the Middle East [34], they are prevalent in other neighbouring countries, namely Turkey [35] and Pakistan [36], as well as the Gulf Cooperation Council (GCC) countries [15]. Two founder-effect mutations, p.Arg1792His and p.Glu57Ter, have been reported in multiple Saudi families with hearing loss. The latter mutation was also found in Arab HL patients from Libya and Tunisia [37, 38]

The pathogenic splice site variant c.5292-2A > C results in an A to C transversion which disrupts the consensus 5’ splice site (acceptor site) located at the intron 42/exon 43 junction of the *OTOF* gene and ultimately leads to aberrant splicing of its pre-mRNA. Consequently, exon 43 may be completely or partly skipped, and/or a cryptic splice site may be used. Our in silico analysis suggests that a truncated protein (1815 amino acids) that lacks the transmembrane (TM) domain and has a partial loss of the C2F domain would be synthesized (Fig. 4C). Multiple short and long *OTOF* isoforms have been described to be important for auditory function [11, 14], and all of them would be impacted by the c.5292-2A > C variant.

Splice site variants have been predicted to cause at least 15% of all human diseases. Currently, the Human Gene Mutation Database (HGMD) (http://www.hgmd.cf.ac.uk/) has splice site variants that account for 8.6% of total variants implicated in various clinical conditions; a percentage that is thought to be an underestimation as aberrant splicing may be the consequence of some deep intronic, exonic sequences, or cis-element sequences that are often undetected by common variant prioritization that focus solely on variants near ± 2 splice sites [39]. To date, 28 pathogenic/likely pathogenic splice site *OTOF* variants have been described in the ClinVar database. However, our variant c.5292-2A > C was absent from this database among other available databases, further suggesting that it is a pathogenic variant in addition to its confirmed segregation with the hearing loss phenotype. In support of this finding, the similar variants c.766-2A > G and C.3571-2A > C located at the splice sites of exon 7 and exon 27, respectively, were reported to be implicated in DFNB9 [11, 40]. Finally, based on the ACMG/AMP guidelines the NM_001287489.2 (*OTOF*):c.5292-2A > C variant met the criteria of PVS1_Strong, PM2_Strong, and PP1_supporting for an overall classification as “pathogenic”.

To the best of our knowledge, *CLDN14* is the only deafness-implicated gene reported in the literature in the Yemeni population [41]. Our findings add the *OTOF* and the *MYO15A* genes as two additional genes responsible for non-syndromic hearing loss in this population.

## Conclusions

Clinical exome sequencing of two unrelated Yemeni families affected with ARNSHL revealed the presence of two novel pathogenic variants, c.6347delA and c.5292-2A, in the *MYO15A* and the *OTOF* genes, respectively. This study supports previously published studies regarding the significant role of these two genes in ARNSHL in Middle Eastern individuals and expands their mutational spectra [14–16, 42–46].

## Data Availability

All data generated or analysed during this study can be obtained from the corresponding author on request.

## Abbreviations

NGS: Next-generation sequencing
MYO15A: Myosin XVA
OTOF: Otoferlin
PCR: Polymerase chain reaction
RFLP: Restriction fragment length polymorphism
HL: Hearing loss
ARNSHL: Autosomal recessive non-syndromic hearing loss
ATP: Adenosine triphosphate
DFNB: Deafness, neurosensory, autosomal recessive
sh2: Shaker-2
BACs: Artificial bacterial chromosomes
CES: Clinical exome sequencing
GJB2: Gap junction protein Beta 2
BWA: Burrows–wheeler aligner
GATK: Genomic analysis toolkit
VariMAT: Variation and Mutation Annotation Toolkit
ExAC: The exome aggregation consortium
GnomAD: Genome aggregation database
dbSNP: Single nucleotide polymorphism database
SIFT: Sorting intolerant from tolerant
VEP: Variant effect predictor
PolyPhen-2: Polymorphism phenotyping v2
BLAST: Basic Local Alignment Search Tool
MYO3A: Myosin IIIA
MYO6: Myosin VI
MYO7A: Myosin VIIA
WHRN: Whirlin
EPS8: Epidermal growth factor receptor pathway substrate 8
PTC: Premature termination codon
NMD: Nonsense-mediated decay
MyTH4-FERM: Myosin tail homology 4-band 4.1, ezrin, radixin, and meosin
SH3: SRC homology 3
DFNA: Deafness, autosomal dominant
USH1B: Usher syndrome 1B
ACMG/AMP: American College of Medical Genetics and Genomics/Association for Molecular Pathology
SGNs: Spiral ganglion neurons
IHC: Inner hair cell
GCC: Gulf Cooperation Council
HGMD: Human Gene Mutation Database
CLDN14: Claudin 14
